# The Role of Biomarkers in Temporomandibular Disorders: A Systematic Review

**DOI:** 10.3390/ijms26135971

**Published:** 2025-06-21

**Authors:** Joana Maria Soares, Bruno Daniel Carneiro, Daniel Humberto Pozza

**Affiliations:** 1 Department of Biomedicine, Unit of Experimental Biology, Faculty of Medicine, University of Porto, 4200-319 Porto, Portugal; joanbsoares@gmail.com (J.M.S.); bcarneiro@med.up.pt (B.D.C.); 2 Rheumatology Service, Unidade Local de Saúde do Alto Minho, Hospital Conde de Bertiandos, 4990-078 Ponte de Lima, Portugal; 3 Institute for Research and Innovation in Health and IBMC, University of Porto, 4200-135 Porto, Portugal

**Keywords:** chronic pain, temporomandibular joint, temporomandibular disorders, biomarkers, inflammatory cytokines, oxidative stress, matrix metalloproteinases

## Abstract

Temporomandibular disorders (TMDs) impact quality of life and present diagnostic and treatment challenges. Biomarkers may serve as an additional tool to support diagnosis and monitor disease progression, offering supplementary information for treatment strategies in specific and selected patients. This systematic review aimed to assess the role of biomarkers in diagnosing TMD and guiding personalized treatment. It also examined key biomarkers linked to chronic temporomandibular joint (TMJ) pain and how therapies affect biomarker levels and clinical outcomes. A comprehensive search was conducted in PubMed, Scopus, and Web of Science to identify observational and interventional studies assessing the role of biomarkers in synovial fluid/tissue, saliva, and blood. The research was registered in PROSPERO, adhered to PRISMA guidelines, and employed Cochrane Risk of Bias tools. To assess the effect, only studies examining biomarker levels were considered. A total of forty-six studies met the inclusion criteria: three randomized controlled trials were rated as having some concerns, as were most of the observational studies. Elevated levels of interleukins (1ß and 6), tumour necrosis factor alpha, and prostaglandin E2 in synovial fluid were correlated with temporomandibular joint (TMJ) inflammation. Increased matrix metalloproteinases (2, 7, and 9) indicated cartilage deterioration, while oxidative stress markers such as malondialdehyde were higher in TMD patients. Treatments including hyaluronic acid, platelet-rich plasma, and low-level laser therapy effectively reduced inflammatory biomarkers and improved symptoms. Biomarkers show potential to contribute to the understanding of pathophysiological mechanisms in TMD and may support future diagnostic and therapeutic strategies for selected patients. After high-quality studies confirm these findings, this approach will enable personalized medicine by tailoring treatments to individual patient profiles, ultimately leading to improved outcomes and quality of life.

## 1. Introduction

Temporomandibular disorders (TMDs) encompass muscular and joint conditions affecting the masticatory muscles, the temporomandibular joint (TMJ) and the associated structures, and represent a heterogeneous group of musculoskeletal conditions—including myofascial pain, arthralgia, and articular disc displacement (DD)—that can lead to degenerative diseases [1]. These changes can impair essential functions such as speech and chewing, potentially causing debilitating pain [2], which is often the primary sign of TMD and the main reason for patients to pursue treatment. The development of degenerative joint diseases can be caused by synovial membrane inflammation. This process of inflammation can trigger a cascade of events, including pain, that result in fibrosis and muscle weakness, leading to the destruction of the articular surfaces and the failure of the lubrification system [2,3].

The classification of chronic pain varies among researchers and healthcare professionals and may be related to central sensitization [4,5]. According to the International Association for the Study of Pain (IASP), pain is considered chronic when it persists beyond the normal healing time, whereas in clinical and research contexts, a period of 3 to 6 months is commonly used for this definition [3]. Chronic pain is a multifactorial phenomenon influenced by biological, psychological, social, and spiritual aspects [6,7]. Approximately 20% to 30% of the global population experiences chronic pain, significantly affecting quality of life [6]. The prevalence of TMD varies widely, affecting between 5% and 12% of the general population, with a higher incidence in women. Chronic orofacial pain is often associated with persistent TMD and may involve factors such as inflammation, structural changes, muscle spasms, or neural sensitization [3,4].

The impact of chronic TMJ pain extends beyond the physical dimension, significantly affecting patients’ quality of life. In addition to persistent pain, it is frequently associated with psychological disorders, autonomic disturbances, and sleep problems [8]. Managing this condition is challenging due to the difficulty of identifying its exact cause. Therefore, treatment focuses on rehabilitation and improving quality of life rather than achieving a definitive cure [4]. The lack of a complete understanding of chronic TMD pathogenesis results in a diagnostic process primarily based on clinical evaluation, interviews, and imaging exams, when appropriate, leading to symptomatic therapeutic approaches rather than treatments targeted at the disease’s pathophysiology [9].

Biomarkers have emerged as a promising alternative to improve the diagnosis and treatment of TMD. Biomarkers are measurable characteristics that indicate normal or pathological biological processes, as well as responses to therapeutic interventions [2]. They can be classified as inflammatory (interleukins, tumour necrosis factor, and prostaglandins), oxidative (molecules associated with oxidative stress), neuropeptidergic (such as substance P and calcitonin gene-related peptide), and cartilage degradation markers (such as matrix metalloproteinases) [2]. These biomarkers can be identified in different sample types, including synovial fluid, saliva, and blood, expanding their potential for clinical use [2].

Given the subjective, complex, and multifactorial nature of chronic TMJ pain, identifying reliable biomarkers is of significant clinical relevance. Biomarkers can provide valuable insights into the underlying pathophysiological mechanisms of pain chronicity in TMD, enabling the earlier and more precise diagnosis, risk stratification, monitoring of disease progression, and assessment of therapeutic response. From a clinical perspective, integrating biomarker profiles into routine assessment could support the development of personalized treatment approaches, optimizing outcomes and reducing the burden of chronic pain. Despite growing interest in the field, the current evidence on biomarkers for chronic TMJ pain remains fragmented and inconclusive, with no clear consensus on their diagnostic, prognostic, or monitoring roles. This lack of clarity makes it difficult to apply the findings in clinical settings and highlights the need for a thorough review of the research [10]. Moreover, previous systematic reviews focused on specific aspects such as biomarkers from saliva [11,12], cortisol levels [13], vitamin D [14], and total antioxidative status [15], highlighting the need for a more integrative approach.

This review seeks to address the existing gap by providing a critical synthesis of the available studies. High levels of pro-inflammatory cytokines were previously reported in conditions such as osteoarthritis (OA) and TMJ internal derangement (TMJ-ID), but inconsistencies in results and a lack of clinical validation remain [2]. Furthermore, the interaction between biomarkers and the various factors influencing chronic TMD pain has not yet been fully elucidated. In this context, this systematic review primarily aimed to evaluate the role of biomarkers in the early diagnosis of TMD and in guiding personalized treatment. Secondary objectives included identifying key biomarkers linked to chronic TMJ pain and examining how treatments influence biomarker levels and clinical outcomes.

## 2. Materials and Methods

According to the Preferred Reporting Items for Systematic Reviews and Meta-Analyses (PRISMA) guidelines, a systematic review was performed [16]. In addition, the review was listed in the “International Prospective Register of Systematic Reviews” (PROSPERO) database protocol as “The Role of Biomarkers in Chronic Pain of the Temporomandibular Joint: A Systematic Review” with the identification number CRD420250650948 to guarantee study transparency and reproducibility. The PICO question for this review was “In individuals with chronic temporomandibular joint pain (P), how do biomarkers associated with the condition (I) compare to the absence of biomarkers or different biomarker profiles (C) in assessing, diagnosing, and predicting disease progression (O)? If they have a significant role in TMJ, which ones do we choose?”

A search, carried out in January 2025, was made in three electronic bibliographic databases, which included PubMed, Web of Science, and Scopus, with no temporal limit. The search strategy was based on the terms “Biomarkers” and “Temporomandibular Joint Disorders”. The specific search strategy in Pubmed was as follows: (“Biomarkers” [Mesh]) AND “Temporomandibular Joint Disorders” [Mesh]; in Web of Science, it was as follows: Biomarkers AND Temporomandibular Joint Disorders (Topic); and in Scopus, it was as follows: biomarkers AND temporomandibular AND joint AND disorders.

The inclusion criteria covered adult humans (≥18 years) diagnosed with chronic non-cancer TMJ pain (pain persisting for ≥3 months); patients with TMD, including OA or other TMJ-related chronic pain conditions; studies assessing biomarkers (e.g., inflammatory, oxidative stress, neuropeptides) in biological samples (saliva, blood, synovial fluid/tissue) related to TMJ pain; observational (cross-sectional, cohort, case–control); and interventional studies (randomized controlled trials—RCTs) investigating biomarkers relevance in chronic TMJ pain. The exclusion criteria comprised studies focused only on acute TMJ pain (<3 months); patients with TMJ pain secondary to systemic diseases (e.g., rheumatoid arthritis, fibromyalgia, systemic lupus erythematosus); studies that did not investigate biomarkers or did not report biomarker-related outcomes; case reports; narrative reviews; and studies with insufficient data on biomarker analysis.

Titles and abstracts were screened independently by two of the authors to assess their relevance and eligibility with the objective of this systematic review in the Rayyan tool online version (Rayyan Systems, Inc., Cambridge, MA, USA, https://rayyan.ai/ accessed on 21 January 2025). The screening was made with the “blind mode” to ensure decision individuality, preserve objectivity, and minimize bias in the process. Afterwards, the two authors worked together to solve the conflicts, reaching full agreement. The level of agreement between the authors was assessed using the Kappa test [17].

A full-text review was conducted for a comprehensive analysis and key information from each selected study was extracted, including authors’ information, publication year, publication country, participants’ characteristics, number of participants, the intervention (in assessing diagnosis or treatment) details, and the main results. The extracted data was then organized into two charts, depending on whether the study focused on diagnosis, treatment, or both. The studies were then systematically evaluated, ensuring that only studies with the appropriate methodology and relevant outcomes were included, guaranteeing the validity and reliability of the results.

The risk of bias of the clinical trials was evaluated with the Cochrane RoB 2 tool [18] at the outcome level visualized with the Cochrane risk of bias VISualization app 4.0 [19] and the risk of bias of other study types was evaluated with the ROBINS-E tool [20], also at the outcome level visualized with the Cochrane risk of bias VISualization app 4.0 [19].

## 3. Results

A comprehensive search of the literature in three databases resulted in 331 potential records being identified: 118 from PubMed, 72 from Web of Science, and 141 from Scopus. Following the removal of duplicate records, 218 manuscripts remained for the title and abstract review. Records were screened, and 164 were excluded after screening by title and abstract. The remaining 54 reports were assessed for eligibility and full-text examination. The final inclusion criteria were met by 46 manuscripts.

Eight articles were excluded due to the following reasons: one did not describe diagnostic or treatment criteria; one included patients under 18 years of age; one involved non-human subjects; one had an inappropriate study design; one used urine samples; one used hair samples; one did not involve chronic pain; and one focused on muscular rather than joint pain. The Kappa coefficient for interrater agreement was 0.84, and the disagreement was resolved by consensus among the three authors.

**Figure 1:**
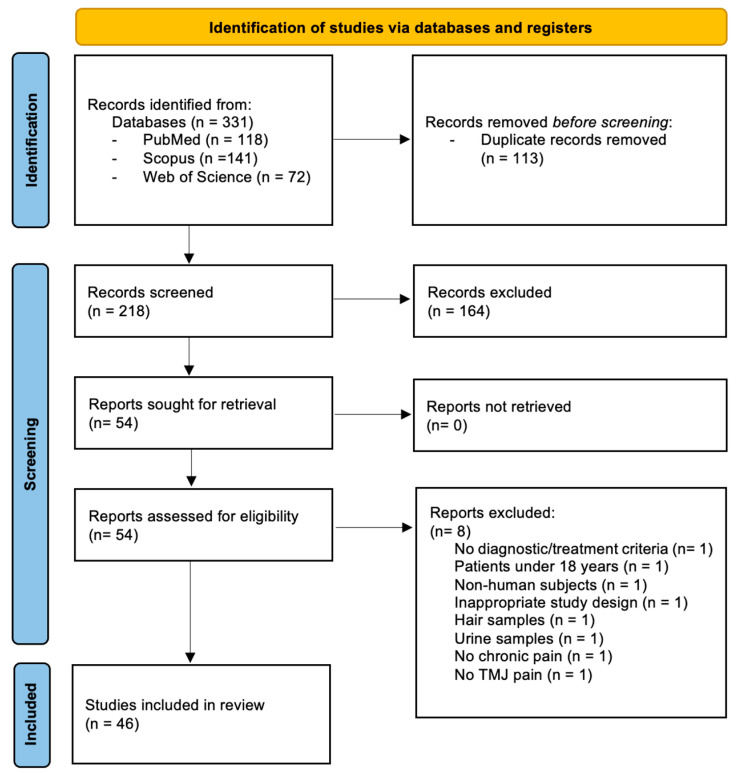
PRISMA flow diagram outlining the selection of the included studies.

The PRISMA flowchart is depicted in Figure 1. The extracted data characteristics for each study are available in Table 1 and Table 2, depending on whether the study focused on diagnosis or treatment, respectively.

### 3.1. Characteristics of Included Studies

This systematic review included a total of 46 studies from 1997 to 2024. These studies were selected based on their relevance to temporomandibular joint disorders and the biomarkers associated with these conditions. These articles were conducted in various countries: 7 in North America (USA, Canada), 24 in Asia (Japan, India, South Korea, China, Iraq, Iran, Saudi Arabia, Taiwan, Indonesia, Malaysia), and 15 in Europe (Turkey, Croatia, Italy, Netherlands, Norway, Sweden).

The study designs varied; the majority (*n* = 30) were cross-sectional [9,22,23,25,26,27,28,31,32,35,36,38,40,42,45,46,47,48,49,50,51,52,53,54,55,56,57,58,59,60], 14 were case–controls [10,21,24,29,30,33,34,37,39,41,42,43,44,57], 3 were RCTs [61,62,63], and 1 was non-RCT [8].

The studies analyzed different types of samples; 25 studies evaluated synovial fluid/tissue [10,26,27,32,34,36,38,41,42,45,46,47,48,49,50,51,52,53,54,55,56,57,58,60,63], 15 studied blood [9,23,25,29,30,31,35,37,39,40,41,43,44,59,61], and 8 studied saliva samples [8,21,22,24,25,28,33,62]. The biomarkers investigated in these samples included cytokines (interleukin (IL)-1β, IL-6, IL-8, tumour necrosis factor (TNF)-α) [22,25,26,31,32,41,42,44,45,46,50,51,56,57,58,59,61,63], matrix metalloproteinases (MMP-1/2/3/7/8/9/10/13) [8,34,41,48,53,54,56], oxidative stress markers (malondialdehyde—MDA, total antioxidant capacity—TAC, and Catalase—CAT) [25,33,39,62], and various other proteins and metabolites [9,21,23,24,27,28,29,30,35,36,37,38,40,43,47,49,52,55,60].

The studies included patients with various TMDs, such as degenerative joint disease (osteoarthritis—OA) [10,26,27,28,30,36,38,41,42,43,47,48,49,50,51,54,56,57,58], disc disorders (disc displacement with reduction—DDwR, and disc displacement without reduction—DDwoR) [8,10,21,25,26,27,28,29,30,32,34,36,38,40,46,47,48,49,50,53,54,56,58,60,63], joint pain (arthralgia) [9,22,26,28,31,35,51,59,62], and hypermobility disorders (subluxations) [29]. The records also assessed different forms of treatments for TMD symptoms such as intramuscular injections of hyaluronic acid (HA) [8,58]; platelet-rich plasma (PPR) [8]; conservative therapy [9,59,60,61], including low-level laser therapy (LLLT) [61], and stabilization splint (SS) [62]; pharmacological interventions, including glucosamine–chondroitin sulphate (GCS), tramadol, and sodium hyaluronic acid (SHA) [63]; and invasive surgical treatment [60].

The mean age of the patients ranged from 18 to 71 years, with a higher prevalence of female patients in most studies. The mean percentage of females over males across the total number of articles was 69.94%.

### 3.2. Summary of Key Findings

Several studies investigated synovial fluid and tissue biomarkers in TMD. Increased levels of IL-1β, IL-6, TNF-α, and PGE2 were reported in synovial fluid samples from patients with TMD, particularly in cases with degenerative joint disease and internal derangement [34,42,58,61]. A study examining the effect of intra-articular HA injections found that levels of high-mobility group box 1 (HMGB1), IL-1β, IL-18, PGE2, toll-like receptor 4 (TLR4), and inducible nitric oxide synthase significantly decreased after treatment [58].

Increased MMPs were also reported. Patients with TMD had higher concentrations of MMP-2 and MMP-9 in synovial tissue, correlating with joint degeneration and inflammatory responses [8,34,48]. Another study found that synovial fluid samples from patients with DDwoR exhibited significantly higher levels of bone morphogenetic protein 4 (BMP-4), eotaxin, and IL-8, particularly in cases with sudden onset symptoms [34].

Additional findings included increased levels of aggrecan and PGE2 in OA cases compared to DDwoR [10,34]. Moreover, a strong correlation was observed between synovial fluid and synovial tissue concentrations of IL-1β, IL-10, and TNF-α in degenerative joint disease [58,61].

Blood-based biomarkers were examined in relation to systemic inflammatory responses and their correlation with pain improvement in TMD patients. Studies reported increased levels of neutrophil-to-lymphocyte ratio (NLR), derived NLR (dNLR), platelet-to-lymphocyte ratio (PLR), systemic immune-inflammation index (SII), and total protein in patients experiencing TMD-related pain [9,59]. In contrast, the lymphocyte-to-monocyte ratio (LMR) was significantly lower in patients with persistent pain [9].

In terms of stress-related biomarkers, higher concentrations of cortisol and norepinephrine were found in the long-sleep-duration group, whereas patients with a short sleep duration exhibited increased inflammatory markers, including IL-1β, IL-4, and IL-8, with a significant association with pain severity [21,59]. Another study identified significantly elevated levels of IL-8, IL-2, IL-13, interferon-gamma (IFN-γ), PGE2, and thrombopoietin in patients with a severe pain disability [59].

A separate study reported increased levels of osteopontin (OPN) in patients with TMJ-ID, while CD44 levels did not show significant differences between TMD and control groups [39]. Furthermore, higher levels of calcitonin gene-related peptide (CGRP) were associated with age and obesity, but no significant correlation was found with clinical TMD characteristics [43].

Salivary biomarkers were examined as potential non-invasive diagnostic tools for TMD. Increased levels of MMP-2 and MMP-9 were detected in the saliva samples of patients with TMJ-ID, which showed a significant reduction following HA and PRP treatment [8]. Additionally, salivary cortisol levels were found to be significantly higher in patients with disc displacement without reduction and limited mouth opening [21].

A study investigating oxidative stress markers found that salivary levels of MDA were significantly elevated in TMD patients compared to controls, whereas TAC levels were lower [33]. Moreover, no significant differences in CAT levels were observed between groups [33].

Saliva C-reactive protein (CRP) and IL-1β levels were analyzed in post-orthodontic patients with TMD symptoms, showing increased expression, although the differences were not statistically significant [22].

The studies reviewed explored different treatment modalities and their impact on biomarker expression in TMD patients. Intra-articular injections of HA and PRP led to a significant reduction in MMP-2 and MMP-9 saliva levels, reflecting a decrease in joint inflammation [8].

LLLT was associated with a decrease in blood IL-6 and C-reactive protein (CRP) levels in patients with painful TMD [61]. Patients who underwent conservative therapy, including physical therapy and occlusal splints, exhibited significant reductions in circulating systemic inflammatory markers such as NLR, dNLR, and SII, although the changes were not statistically significant [9].

Pharmacological interventions, such as GCS and tramadol, were found to reduce IL-1β, TNF-α, and PGE2 synovial levels, while IL-6 levels increased in certain treatment groups [63].

Stabilization splints were reported to decrease saliva oxidative stress markers, including superoxide dismutase (SOD) and TAC, in patients with TMJ arthralgia [62]. Furthermore, the erythrocyte sedimentation rate (ESR) was significantly associated with pain improvement at three months post-treatment [9].

Correlations between biomarker levels and clinical parameters were also observed. A positive correlation was identified between salivary MMP-2 and MMP-9 levels, pain severity, and joint clicking, while a negative correlation was found with maximum mouth opening (MMO) [8]. Similarly, IL-1β, IL-18, and TLR4 synovial levels were positively correlated with inflammation severity [58].

In synovial fluid samples, a negative correlation was reported between complement factor H-related protein 3 (CFHR3) and pain levels, while radixin (RDX) and carboxy-peptidase N catalytic chain (CPN2) exhibited a positive correlation with pain severity [59].

Elevated levels of IL-8, IL-2, and IL-13 in blood samples were associated with reduced jaw function and higher generalized pain intensity [59]. Additionally, norepinephrine levels were significantly lower in the long-sleep-duration group, while ESR levels were correlated with significant pain improvement at three months post-treatment [9].

### 3.3. Risk of Bias

**Figure 2:**
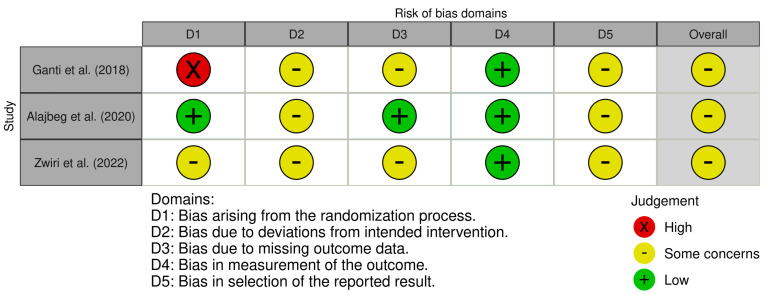
Risk of bias, represented in five categories and with its overall result, for each randomized clinical trial included in the review: Zwiri, A.M. et al. (2022) [61], Alajbeg, I.Z. et al. (2020) [62], Ganti, S. et al. (2018) [63].

**Figure 3:**
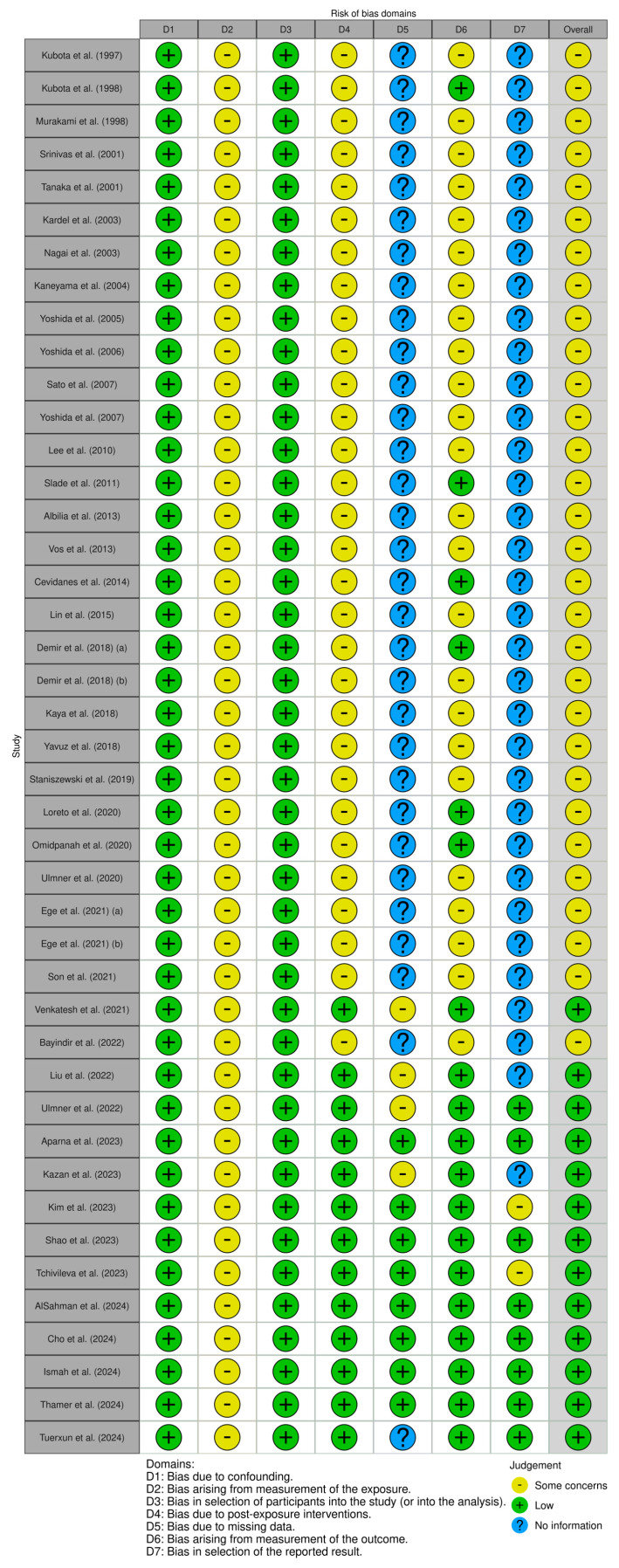
Risk of bias, represented in seven categories and with its overall result for each observational study included in the review: Thamer, S.R. and Diajil, A.R. (2024) [8], Cho, I.S. et al. (2024) [9], Shao, B. et al. (2023) [58], Kim, Y. et al. (2023) [59], Liu, X. et al. (2022) [60], Tuerxun, P. et al. (2024) [10], AlSahman, L. et al. (2024) [21], Ismah, N. et al. (2024) [22], Tchivileva, I.E. et al. (2023) [23], Aparna, N. et al. (2023) [24], Kazan, D. et al. (2023) [25], Ulmner, M. et al. (2022) [26], Bayındır, S. et al. (2022) [27], Venkatesh, S.B. et al. B. et al. (2021) [28], Ege, B. et al. (2021) [29], Ege, B. et al. (2021) [30], Son, C. et al. (2021) [31], Ulmner, M. et al. (2020) [32], Omidpanah, N. et al. (2020) [33], Loreto, C. et al. (2020) [34], Staniszewski, K. et al. (2019) [35], Yapıcı, Y.G. et al. (2019) [36], Demir, C.Y. et al. (2018) [37], Kaya, G.S. et al. (2018) [38], Demir, C.Y. et al. (2018) [39], Lin, S.L. et al. (2015) [40], Cevidanes, L.H. et al. H. et al. (2014) [41], Vos, L.M. et al. (2013) [42], Albilia, J.B. et al. (2013) [43], Slade, G.D. et al. D. et al. (2011) [44], Lee, J.K. et al. (2010) [45], Sato, J. et al. (2007) [46], Yoshida, H. et al. (2007) [47], Yoshida, K. et al. (2006) [48], Yoshida, K. et al. (2005) [49], Kaneyama, K. et al. (2004) [50], Kardel, R. et al. (2003) [51], Nagai, H. et al. (2003) [52], Srinivas, R. et al. (2001) [53], Tanaka, A. et al. (2001) [54], Murakami, K.I. et al. (1998) [55], Kubota, E. et al. (1998) [56], Kubota, E. et al. (1997) [57].

The graphical representations of the risk of bias of the analyzed studies are shown in Figure 2 and Figure 3. Regarding the randomized controlled trials (Figure 2), the three studies were classified as having some concerns—moderate risk of bias [61,62,63]. For the observational studies (Figure 3), thirteen presented a low risk of bias; however, most studies were classified as having a moderate risk of bias, with domains such as selection of participants and measurement of outcomes frequently showing concerns. Several studies also had domains with unclear information, particularly concerning missing data and selection of reported results, which limited the overall assessment of bias.

## 4. Discussion

This systematic review highlights the growing importance of biomarkers in understanding the pathophysiology, diagnosis, and treatment of TMD. The results confirming inflammatory cytokines, oxidative stress markers and MMPs are consistently associated with the severity and progression of TMD. These findings suggest that biomarkers can play a crucial role in the early identification of patients at risk for chronic disease progression, thus improving diagnostic accuracy and enabling more targeted therapeutic interventions.

The involvement of inflammatory cytokines in TMD has been extensively documented. Elevated levels of IL-1β, IL-6, TNF-α, and PGE2 in synovial fluid and associated tissues suggest that chronic inflammation plays a key role in disease development [26,41,46,50,56,58]. These cytokines contribute to an inflammatory cascade driving joint degeneration and pain sensitization. IL-1β plays a key role in TMD pathophysiology by influencing pain and cartilage damage, while elevated synovial IL-6 and TNF-α levels in symptomatic patients underscore their involvement in local inflammation [22,32]. MMP-2 and MMP-9 are linked to extracellular matrix breakdown and cartilage damage [8,34,48,49,53,54]. With age, MMP-2 decreases while MMP-9 increases, indicating a shift toward pathological remodelling [64]. Elevated salivary MMP-9 in TMD patients suggests its potential as a non-invasive biomarker, though age-related variations must be considered [65]. Therefore, factors such as age, along with other individual variables like sex, systemic health, and oral hygiene, should be considered when interpreting salivary biomarkers levels to ensure accurate clinical assessment.

Oxidative stress plays a key role in the pathology of TMD, resulting from an imbalance between oxidative damage and the body’s antioxidant defences [33]. This contributes to inflammation, tissue damage, and pain [55]. Higher levels of free radicals and inflammatory mediators in TMD synovial fluid further support this association [66], and ongoing joint inflammation and hypoxia may worsen oxidative damage and disease progression [67]. Blood biomarkers were also found to be elevated in painful TMP, supporting the hypothesis that systemic inflammation plays a role in disease manifestation [9,23,59,61,68]. Notably, hematologic markers vary based on ethnicity and gender, suggesting that establishing population-specific reference values is necessary for accurate interpretation [69]. One advantage of understanding these mechanisms is the potential for targeted therapies that can mitigate inflammation and alleviate symptoms. However, the complexity of these molecules’ interactions makes it challenging to develop effective treatments without unintended effects.

Furthermore, chronic pain starts with peripheral sensitization, being dependent on the reported biomarkers. This inflammation stimulates the central nervous system and can lead to chronic pain. Thus, early diagnosis using blood or saliva can be of extreme importance to avoid disease progression, which can lead to very difficult-to-manage symptoms, as well as degenerative processes that will reduce the quality of life [68,70,71].

While synovial fluid provides precise insights into the local environment of the TMJ and allows for the targeted detection of inflammatory markers specific to TMD, its invasive collection and need for specialized techniques limit its routine clinical use [26,27,36,38,48,58,72]. In contrast, blood and saliva offer more accessible and minimally or non-invasive alternatives. Blood tests can reflect systemic inflammation and are widely available. However, they may lack specificity for localized joint conditions and can be influenced by individual factors such as ethnicity and gender [23,25,29,30,31,35,39,40,73]. Saliva, due to its non-invasive and convenient nature, is particularly promising for detecting TMD-related biomarkers such as cortisol, matrix metalloproteinases, oxidative stress markers, and stress-related mediators like norepinephrine. These salivary biomarkers can reflect both local and systemic inflammation, correlate with disease severity and sleep disturbances, and provide insights into hypothalamic–pituitary–adrenal axis dysregulation. However, their diagnostic accuracy may be affected by factors like hydration and oral health [12,21,25,28,40,59,74,75]. Future research should prioritize standardizing protocols and precise recommendations for biomarker analysis to enhance reproducibility and clinical utility across diverse settings.

Several therapeutic strategies have been evaluated based on their effects on biomarker levels. Intra-articular injections of HA and PRP have shown promise in reducing the biomarker levels, pain, and jaw dysfunction scores, offering potential benefits in modulating joint degeneration [8,58]. Additionally, HA administration was found to prolong its retention in the articular space, enhancing its therapeutic efficacy [76]. However, these infiltrations are not long-lasting, and if the cause of the degenerative process is not addressed, the inflammatory process will return. Conservative treatments, including occlusal splints, physical therapy, and behavioural interventions, have also been associated with reductions in inflammatory biomarkers and improvements in pain symptoms [9,59]. Studies also support the efficacy of LLLT in reducing IL-6 and high-sensitivity (hs)-CRP levels, strengthening its anti-inflammatory properties [61].

Other pharmacological treatments, such as glucocorticoids and tramadol, have been found to significantly reduce IL-β, TNF-α, and PGE2 levels, encouraging their potential role in cytokine modulation [38]. However, it remains unclear whether these treatments alter disease progression or merely provide symptomatic relief. Notably, the selective inhibition of HMGB1 has been shown to exert chondroprotective effects by blocking IL1-induced MMP expression, emphasizing a feasible therapeutic target for TMD [77]. The use of chronic pharmacological treatments, such as glucocorticoids and opioids, has some adverse effects that should be considered. Glucocorticoids can lead to side effects like osteoporosis, weight gain, mood swings, and an increased risk of infections. Opioids may cause drowsiness, constipation, nausea, and, more seriously, physical dependence, addiction, and overdose. These adverse effects highlight the importance of careful management and monitoring when using these medications for chronic conditions [78,79]. In this context, less invasive treatments may be preferred over irreversible approaches, particularly when managing early-stage TMD, as the primary strategy should be a conservative, multimodal treatment plan tailored to the patient’s specific complaints and characteristics. The focus should remain on symptom control and functional recovery [80].

The integration of biomarker profiling into clinical practice holds significant promise for advancing the diagnosis, prognosis, and management of TMD. Salivary and serum biomarkers such as cortisol, IL-8, IL-1β, TNF-α, and oxidative stress markers have demonstrated potential in differentiating TMD subtypes, monitoring disease progression, and evaluating treatment response [59,61,62,63]. Hematologic indicators, including hemoglobin and lymphocyte-to-monocyte ratios, may serve as prognostic tools to predict long-term treatment outcomes [9,59]. Moreover, the observed associations between psychological factors, such as stress and depressive symptoms, and biomarkers like cortisol and oxidative stress markers, underscore the importance of psychosomatic integration in TMD care [8,24,25,28,40,62]. Molecular profiling approaches that combine clinical and biochemical data offer deeper insights into TMD pathogenesis and may facilitate the development of precision medicine strategies. Additionally, biomarkers can help in real-time treatment adjustments, enhancing therapeutic efficacy.

Despite the promising implications of biomarker research in TMD, some limitations must be acknowledged. Many of the included studies were observational, which restricts the ability to establish causal relationships between the biomarker levels and disease progression. Most available studies evaluate biomarkers at a single time point, which precludes their application as reliable prognostic tools for chronic pain development or treatment response in TMD. Additionally, the lack of standardization in biomarker measurement techniques across studies complicates direct comparisons and meta-analyses.

Future well-designed, longitudinal, and interventional studies are needed to validate the predictive value of specific biomarkers, establish standardized protocols for their assessment, and integrate them with comprehensive pain and functional outcome measures. Pain should be more thoroughly assessed and monitored over time to better understand its evolution and correlation with clinical and biological indicators. Likewise, the heterogeneity in the study populations, including the differences in sex distribution, ethnic background, and disease severity, underscores the need for personalized approaches to biomarker analysis. Overall, the choice of diagnostic method should consider the balance between precision, invasiveness, and accessibility. Studies evaluating sex-based differences in biomarker expression could provide valuable insights, as hormone fluctuations may influence inflammatory and oxidative stress responses in TMD [69].

The integration of machine learning and artificial intelligence (AI) in biomarker analysis holds significant promise for improving predictive modelling and enabling more accurate disease classification in TMD. The inclusion of antibody profiles and genetic data can further strengthen diagnostic precision by offering complementary insights into underlying pathophysiological mechanisms [68,81]. Future developments may further benefit from combining AI with biosensor technologies, enhancing the diagnostic potential of biomarker research through real-time, non-invasive monitoring. For example, it the feasibility of sensor-based tracking of temporomandibular function and the application of machine learning models to predict biomarkers in pain-related conditions were demonstrated [82]. These advances underscore the value of interdisciplinary approaches in advancing personalized diagnostics and individualized pain management strategies in TMD care.

## 5. Conclusions

This systematic review underscores the promising role of biomarkers in the diagnostic, prognostic, and treatment management of chronic pain and disease progression in TMD patients. Inflammatory cytokines, oxidative stress markers, and MMPs are consistently associated with disease severity and progression, highlighting their potential as diagnostic and therapeutic targets. Treatments such as HA and PRP injections and conservative therapies show efficacy in modulating biomarker levels and improving clinical outcomes. However, the standardization of biomarker assessment methodologies and further validation through randomized controlled trials are necessary to establish their clinical utility. Ultimately, integrating biomarkers into a personalized medicine approach may revolutionize the management of TMD, improving patient outcomes and guiding future research.

## Figures and Tables

**Table 1:** Comparative overview of the key characteristics of the included studies on temporomandibular disorders, focused on the diagnosis.

Reference	Study Type/Country	Type of Sample	Biomarkers	Population Characteristics/Diagnosis	Results
Tuerxun, P. et al. (2024) [10]	Case–control Observational China	Synovial fluid	46 metabolites (fatty/organic/amino acids, sugars, amines, and others)	11 females (41.91± 16.6 years) and 1 male (20 years) TMD: OA or DDwR, pain intensity not reported	OA showed distinct metabolic profiles from DDwR, with L-carnitine, taurine, and adenosine identified as potential biomarkers. TCA cycle and ferroptosis: OA pathogenesis and therapeutics.
AlSahman, L. et al. (2024) [21]	Case–control Observational Saudi Arabia	Saliva	Cortisol	132 patients divided in two groups (TMD: DDwoR vs. control), 18–40 years, pain intensity ≥ 4/10	↑ cortisol: Biomarker for specific TMD subtypes, especially in males with DDwoR.
Ismah, N. et al. (2024) [22]	Cross-sectional Observational Indonesia	Saliva	IL-1β and C-reactive protein (CRP)	77 females and 28 males, 26.4 years TMD with arthralgia, pain intensity not reported	Pain-related or joint TMDs with ↑ CRP > IL-1β. Both types were combined ↑ CRP < IL-1β.
Tchivileva, I.E. et al. (2023) [23]	Cross-sectional Observational USA	Blood	Calcitonin gene-related peptide (CGRP)	80 participants from 18 to 64 years Painful TMDs Average pain intensity of 53.2/100	CGRP associated with age and Body Mass Index, but not chronic painful TMD.
Aparna, N. et al. (2023) [24]	Case–control Observational India	Saliva	Cortisol	50 patients divided in two groups, 18–45 years Painful or symptomatic TMD, pain intensity not reported	No statistically significant difference in salivary cortisol level between cases and controls.
Kazan, D. et al. (2023) [25]	Cross-sectional Observational Turkey	Saliva, blood	IL-6, MDA, 8-OHdG	44 patients, 14–40 years 27 with DDwR/DDwoR vs. 17 controls, pain intensity not reported	Strong positive correlation between pain, 8-OHdG, and IL-6.
Ulmner, M. et al. (2022) [26]	Cross-sectional Observational Sweden	Synovial fluid/tissue	ILs, TNF-α	101 patients, average age of 40.6 years TMD: DDwR, DDwoR, DJD, arthralgia Average pain intensity of 4/10	IL-1β and TNF-α were significantly associated with TMJ palpation pain. TNF-α also correlated with subjective TMJ pain. IL-1β was linked to synovitis, which contributes to pain.
Bayındır, S., et al. (2022) [27]	Cross-sectional Observational Turkey	Synovial fluid	Aggrecan, adiponectin, resistin, apelin, VEGF, and PGE2	41 patients, 12–72 years TMD: DDwR, DDwoR and OA Average pain intensity of 6/10	Aggrecan and PGE2 are linked to localized TMJ pain and are elevated in joints with degenerative changes.
Venkates h, S.B. et al. B. et al. (2021) [28]	Cross-sectional Observational India	Saliva	Cortisol	187 females and 161 males, 18–23 years (20 with TMD vs. 20 controls) TMD: DDwR, DDwoR, arthralgia, and DJD, pain intensity not reported	Cortisol: strong association with stress and TMD severity.
Ege, B. et al. (2021) [29]	Case–control Observational Turkey	Blood	OPN, CD44	71 patients, 18–57 years (54 with TMD vs. 17 controls) TMD: ID and subluxations, qualitative pain evaluated	↓ OPN in TMD patients CD44 no statistical difference.
Ege, B. et al. (2021) [30]	Case–control Observational Turkey	Blood	Asporin	43 controls (31.30 ± 7.53) vs. 43 TMD (31.42 ± 13.24) TMD: DDwoR and OA, pain intensity not reported	Asporin significantly upregulated in TMD.
Son, C. et al. (2021) [31]	Cross-sectional Observational Republic of Korea	Blood	ILs, IFN-γ, TNF-α, growth factors, PGE2, and THPO	66 female participants (24.83 ±3.03 years) TMD with arthralgia Average pain intensity of 5.44/10	TMD—higher pain intensity/duration and ↑ IL-8 and IgG levels link chronic pain and systemic inflammation.
Ulmner, M. et al. (2020) [32]	Cross-sectional Observational Sweden	Synovial tissue	BMP, Epidermal Grow Factor (EGF), ILs and OPG, IFN-γ, IP, eotaxin	51 females and 12 males (41.3 ± 15.1 years) TMD: DDwR (19 patients), DDwoR (44 patients) Pain intensity ≥ 4/10 (DDwoR>DDwR)	DDwoR: IP ↓, OPG ↓ EGF + IL-1 ra ↑ (female > male) sudden onset >delayed onset: BMP 4 ↑, Eotaxin ↑, IL-8 ↑.
Omidpan ah, N. et al. (2020) [33]	Case–control Observational Iran	Saliva	MDA, TAC, and Catalase	30 patients with TMD (30.7 ± 13.2 years) vs. 30 controls (29.16 ± 11.2 years) Painful TMD, pain intensity not reported	TMD: higher MDA levels, no changes in TAC and Catalase.
Loreto, C. et al. (2020) [34]	Case–control Observational Italy	Synovial tissue	MMPs	20 TMD vs. 10 controls DDwoR, pain intensity not reported	MMP-7 and MMP-9 overexpressed in DDwoR.
Staniszewski, K. et al. (2019) [35]	Cross-sectional Observational Norway	Blood	Hemoglobin, cobalamin, albumin, PTH, vit D, creatinine, and potassium	60 patients with TMD vs. 60 controls 20–69 years, mean age 45 years TMD with arthralgia, pain intensity not reported	Serum markers, including vitamin D, were not reliable for TMD diagnosis.
Yapıcı, Y.G. et al. (2019) [36]	Cross-sectional Observational Turkey	Synovial fluid	Visfatin	60 individuals (26.55 ± 8.3 years) with DDwoR and OA Pain intensity > 6/10	↑ Vistafin (OA) Positive correlation between pain and visfatin levels.
Demir, C.Y. et al. (2018) [37]	Case–control Observational Turkey	Blood	25(OH) vitamin D, PTH, calcitonin, calcium, phosphorus, magnesium	50 TMD vs. 50 controls, mean age of 50 years, pain intensity not reported	25(OH) vitamin D, calcitonin, calcium, magnesium, or phosphorus (no differences) ↑ PTH.
Kaya, G.S. et al. (2018) [38]	Cross-sectional Observational Turkey	Synovial fluid	Chemerin	60 patients (26,55± 8,3 years), 16–52 years TMD: ID and OA Average pain intensity of 70/100	Positive correlation between pain and chemerin levels.
Demir, C.Y. et al. (2018) [39]	Case–control Observational Turkey	Blood	MDA, Catalase superoxide dismutase (SOD), GSH	32 patients TMD vs. and 32 controls, aged 16–50 years, pain intensity not reported	TMD: higher MDA and lower Catalase, SOD, GSH), no influence from age or gender.
Lin, S.L., et al. (2015) [40]	Cross-sectional Observational Taiwan	Blood	Cortisol	60 DDwoR patients, 37.7 ± 17.22 years vs. 80 patients DD 36.4± 13.08 years, pain intensity not reported	↑ Cortisol in DDwoR: clinical indicator for distinguishing disc displacement disorders.
Cevidanes, L.H. et al. H. et al. (2014) [41]	Case–control Observational USA	Synovial fluid and blood	MMPs, TIMPs, and several others	24 females (39.9 ± 16 years) 12 OA (47.4 ± 16.1 years) vs. 12 controls (41.8 ± 12.2 years), pain intensity not reported	OA showed bone resorption: ANG and MMPs linked to bone apposition, while IL-6 and TNFα linked to bone resorption.
Vos, L.M. et al. (2013) [42]	Cross-sectional case–control Observational Netherlands	Synovial fluid	Collagen type I/II, IL-1β, TNF-α, PGE2	30 OA patients (9 males, 21 females; 40.1 ± 15.3 years) vs. 10 controls (5 males, 5 females; 30.3 ± 10.8 years), pain intensity not reported	High collagen-II levels suggest it may be a useful marker for cartilage degradation.
Albilia, J.B. et al. (2013) [43]	Case–control Observational Canada	Blood	BMPs, Alpha-2-heremans-schmid glycoprotein (AHSG)	30 patients with DJD (hip patients— 64.6 ± 12.1, TMJ patients—41.6 ± 9.8) vs. 120 controls (mean age 38.8 years) Average pain intensity of 6.6/10	↑ BMP-2, BMP-4, ↓ AHSG levels. These markers may help guide treatment decisions.
Slade, G.D. et al. D. et al. (2011) [44]	Case–control Observational USA	Blood	MCPs, MIPs, ILs, and several others	344 females, 18–60 years TMD, pain intensity not reported	Localized TMD linked to IL-1ra and widespread TMD linked to IL-8. Positive correlation between pain intensity and MCP-1 levels.
Lee, J.K. et al. (2010) [45]	Cross-sectional Observational USA	Synovial fluid	TNF-α and IL-6	24 TMD vs. 5 controls TMD symptomatic, pain intensity not reported	↑ TNF-α and ↑ IL-6 in TMD without significant correlation.
Sato, J. et al. (2007) [46]	Cross-sectional Observational Japan	Synovial tissue	IL-8	44 patients, 6 males and 38 females (mean age of 43 years, 17–84 years), with DDwoR vs. 7 controls Average pain intensity of 6/10	↑ IL-8 in TMD, no significant link to pain or inflammation severity.
Yoshida, H. et al. (2007) [47]	Cross-sectional Observational Japan	TMJ specimens	CD34	20 DD and OA patients vs. 10 controls, 20–72 years, pain intensity not reported	↑ CD34 in TMJ internal derangement linked to angiogenesis.
Yoshida, K. et al. (2006) [48]	Cross-sectional Observational Japan	Synovial fluid	MMPs and aggrecanase	35 patients (17–74 years, mean 36.6 years) with DDwR, DDwoR, and OA vs. 10 controls (16–44 years, mean 23.1 years) Average pain intensity of 60/100 (DDwR), 63.5/100 (DDwoR), and 65/100 in (OA)	↑ MMP-9 in severe TMJ OA and disc displacement. ↑ MMP-2 and aggrecanase were elevated in early OA. Aggrecanase—marker for cartilage degradation.
Yoshida, K. et al. (2005) [49]	Cross-sectional Observational Japan	Synovial fluid	Aggrecanase	35 patients (17–74 years, mean 36.6 years) with TMD vs. 10 controls (16–44 years, mean 23.1 years) TMD: DDwR, DDwoR, and OA Average pain intensity of 61.7/100	↑ Aggrecanase in TMD, especially in severe OA and disc displacement. Aggrecanase—marker for cartilage degradation.
Kaneyama, K. et al. (2004) [50]	Cross-sectional Observational Japan	Synovial fluid	ILs	61 patients (52 females and 9 males) with DDwoR and OA vs. 7 controls, pain intensity not reported	↑ IL-6 and ↑ IL-11 in joints with condylar bone changes: osseous degeneration.
Kardel, R. et al. (2003) [51]	Cross-sectional Observational Sweden	Synovial tissue	ILs, TNF-α, IFN-γ, TGF-β1,2,3, CD68, CD45RO, proliferating cell nuclear antigen	39 patients (19 with arthralgia: 18–66 years and 20 with OA: 26–62 years) Average pain intensity of 5.6/10 in painful clicking and 6.7 in OA	OA joints: ↑ IL-1α, ↑ IL-1β, ↑ IFN-γ, ↑ IL-1ra, ↑ CD68+ macrophages, ↑ inflammation, and ↑ immune activity.
Nagai, H. et al. (2003) [52]	Cross-sectional Observational Japan	Synovial tissue	iNOS, Fas, CD68, and ssDNA	33 patients with TMD (ID and OA), 17–75 years vs. 33 controls, 17 to 54 years old, pain intensity not reported	↑ iNOS, ↑ CD68, ↑ Fas, ↑ ssDNA were linked to synovial changes in TMD disease progression.
Srinivas, R. et al. (2001) [53]	Cross-sectional Observational Canada	Synovial fluid	MMPs	44 TMD with ID patients (33 females and 11 males) with a mean age of 36 years (16–76 years), pain intensity not reported	↑ MMP-1, ↑ MMP-2, ↑ MMP-8, ↑ MMP-9 ↑, MMP-13 in mild TMJ-ID—active collagen degradation.
Tanaka, A. et al. (2001) [54]	Cross-sectional Observational Japan	Synovial fluid	MMPs	38 DDwR, DDwoR, and OA patients, 15–69 years (34.8 ± 14.7 years), vs. 20 controls, 22 to 47 years (26.8 ± 3.7 years), pain intensity not reported	↑ MMP-2 and ↑ MMP-9 in DDwoR>DDwR—diagnostic markers.
Murakami, K.I. et al. (1998) [55]	Cross-sectional Observational Japan	Synovial fluid	PGE2, HA, C4S, and C6S	15 females with painful TMD, mean age of 36.7 years Average pain intensity from 5.1/10 to 6.6/10	↑ PGE2 linked to pain scores ↑ C4S and ↑ C6S linked to TMJ degeneration—markers of proteoglycan breakdown pain-related joint changes.
Kubota, E. et al. (1998) [56]	Cross-sectional Observational Japan	Synovial fluid	MMPs and ILs	22 DDwoR and OA patients vs. 11 controls, pain intensity not reported	IL-1β ↑ (DDwoR), IL-1β ↑ (OA), IL-6 ↑ (OA) —catabolic markers linked to cartilage degradation and pain in TMD.
Kubota, E. et al. (1997) [57]	Case–control Observational USA	Synovial fluid	IL-1β, MMP, TNF-α	22 TMD with OA patients, 15–77 years vs. 15 controls, 18–66 years, pain intensity not reported	↑ IL-1β in TMD (osteolytic changes TMJs) ↑ MMP-3 linked to cartilage degradation —early markers of TMJ deterioration and pain.

**Legend**: ↑: increased, ↓: reduced, BMP: Bone morphogenetic protein, C4/6S: Chondroitin 4/6 sulphate, CD: Cluster of differentiation, CL: Closed lock, DDwR: Disc displacement with reduction, DDwoR: Disc displacement without reduction, DJD: Degenerative joint disease, GSH: Glutathione, HA: Hyaluronic acid, HMGB1: High-mobility group box 1, IFN-γ: Interferon-gamma, IL: Interleukin, iNOS: Inducible nitric oxide synthase, MDA: Malondialdehyde, MIP: Macrophagy inflammatory protein, MMP: Matrix metalloproteinase, OA: Osteoarthritis, OPG: Osteoprotegerin, OPN: Osteopontin, PGE2: Prostaglandin, PTH: Parathyroid hormone, RAGE: Receptor for advanced glycation end-products, TAC: Total antioxidant capacity, TCA: Tricarboxylic acid, THOP: Thrombopoietin, TIMP: Tissue inhibitor of metalloproteinases, TLR4: Toll-like receptor 4, TNF: Tumour necrosis factor, VEGF: Vascular endothelial growth factor.

**Table 2:** Comparative overview of the key characteristics of the included studies on temporomandibular disorders, focused on treatment.

Reference	Study Type/ Country	Type of Sample	Biomarkers	Population Characteristics/Treatment	Results
Thamer, S.R. and Diajil, A.R. (2024) [8]	Non-RCT Iraq	Saliva	Matrix metalloproteinases (MMPs)	32 females and 20 males, 18–55 years with TMD resistant to conservative therapy Intra-articular HA (30 patients) and PRP (22 patients) treatment	MMP-2 and MMP-9 positively correlated with pain and joint click/negatively with mouth opening. HA and PRP therapies reduce inflammation and improve TMD symptoms.
Cho, I.S. et al. (2024) [9]	Cross-sectional Observational South Korea	Blood	Total protein, neutrophils, lymphocytes, monocytes, platelets, and ratios	154 patients, 30.2 ± 10.6 years TMD with arthralgia Average pain intensity of 4/10	69.5% showed significant pain improvement. Hematologic markers, particularly low hemoglobin, may help predict long-term treatment outcomes in TMD.
Shao, B. et al. (2023) [58]	Cross-sectional Observational China	Synovial fluid	HMGB1, interleukins (ILs), PGE2, RAGE, TLR4, and iNOS	Two TMD groups: OA: 77% females, 40.36 ± 9.67 years ID: 70% females, 31.5 ± 10.62 years Intra-articular HA injection 1x/week for 2 weeks Average pain intensity of 5.97/10 in OA and 3.6/10 in DD	High levels of biomarkers, OA > DD HMGB1 levels, pain scores, and jaw dysfunction scores improved after HA treatment.
Kim, Y. et al. (2023) [59]	Cross-sectional Observational South Korea	Blood	ILs, ESR, high-sensitivity C-reactive protein (hs- CRP), cortisol, ACTH, norepinephrine, and epinephrine.	63 females, 24.84 ± 3.00 years, TMD with arthralgia Conservative treatment Hematological analysis at 3 and 6 months post-treatment.	Significant pain improvements (≥2/10) of 64.29%, 41.67%, and 66.67% in normal-, short-, and long-sleep group, respectively. ↑ IL-1β, ↑ IL-4, ↑ IL-8, and ↑ IL-17 showed sufficient strength in predicting significant pain improvement with long-term TMD treatment.
Liu, X. et al. (2022) [60]	Cross-sectional Observational China	Synovial fluid	1714 proteins in the cytosol (43%), plasma membrane (31%), and extracellular space (25%)	95 females and 14 males, 21.31 ± 7.95 years with TMD DDwoR Conservative treatment or disc reposition	↑ ACACB during pain, ↑ HADHA in bruxism, ↑ TGFB1-impaired bone formation. Higher pain levels related to ↑ radixin, ↑ LCP1, ↑ CPN2, ↓ CFHR3, ↓ Factor 11, ↓ INADL, ↓ MBL2.
Zwiri, A.M. et al. (2022) [61]	RCT Malaysia	Blood	hs-CRP, ILs	12 males and 20 females, 20.9 years with painful TMD Conservative treatment (CT), low-level taser therapy (LLLT), and a combination of both	IL-8 may serve as a potential biomarker for TMJ pain: hs-CRP ↑ (LLT, C) ≈ (CT) IL-6 ↑ (LLLT, CT) ↓ (C) IL-8 ↓ (LLLT) ↑ (CT, C); no significant correlation between pain intensity and biomarker levels except for IL6 at baseline and after treatment.
Alajbeg, I.Z. et al. (2020) [62]	RCT Croatia	Saliva	Oxidative stress	20 females, 36.1 ± 11.95 years, TMD with arthralgia Oral splint or placebo for 6 months Average pain in the last 10 days > 30/100	Splint improved pain and depressive symptoms in TMD with associated reductions in oxidative stress.
Ganti, S. et al. (2018) [63]	RCT India	Synovial fluid	ILs, TNF-α, PGE2	30 males and 30 females, 20.9 years with DDwR Treated with glucosamine–chondroitin sulphate, tramadol, or sodium hyaluronate	Treatments improved mouth opening and pain in all groups, with associated reductions in inflammatory markers IL-1β, TNF-α, PGE2, and IL-6.

**Legend**: ↑: increased, ↓: reduced, ACACB: Acetyl-CoA carboxylase beta, ACTH: Adrenocorticotropic hormone, CFHR3: Complement factor H-related protein 3, CPN2: Carboxypeptidase N catalytic chain, DDwR: Disc displacement with reduction, DDwoR: Disc displacement without reduction, ESR: Erythrocyte sedimentation rate, HA: Hyaluronic acid, HADHA: Hydroxyacyl-CoA dehydrogenase trifunctional multienzyme complex subunit alpha, HMGB1: High-mobility group box 1, INADL: inaD-like protein, iNOS: Inducible nitric oxide synthase, LCP1: Lymphocyte cytosolic protein, OA: Osteoarthritis, PGE2: Prostaglandin, PRP: Platelet-rich plasma, RAGE: Receptor for advanced glycation end-products, TGFB1: Transforming growth factor beta 1, TLR4: Toll-like receptor 4, TNF: Tumour necrosis factor.

## Data Availability

All data generated during this study is available upon reasonable request from the corresponding author.

## Abbreviations

The following abbreviations are used in this manuscript:

BMP	Bone morphogenetic protein
CAT	Catalase
CGRP	Calcitonin gene-related peptide
CFHR3	Complement factor H-related protein 3
CPN2	Carboxypeptidase N catalytic chain
CRP	C-reactive protein
DD	Disc displacement
DDwR	Disc displacement with reduction
DDwoR	Disc displacement without reduction
dNLR	Derived NLR
ESR	Erythrocyte sedimentation rate
GCS	Glucosamine–chondroitin sulphate
HA	Hyaluronic acid
HMGB1	High-mobility group box 1
IASP	International Association for the Study of Pain
IFN-γ	Interferon-gamma
IL	Interleukin
iNOS	Inducible nitric oxide synthase
LLLT	Low-level laser therapy
LMR	Lymphocyte-to-monocyte ratio
MDA	Malondialdehyde
MMP	Matrix metalloproteinase
MMO	Maximum mouth opening
NLR	Neutrophil-to-lymphocyte ratio
OA	Osteoarthritis
OPN	Osteopontin
PGE2	Prostaglandin E2
PLR	Platelet-to-lymphocyte ratio
PRISMA	Preferred Reporting Items for Systematic Reviews and Meta-Analyses
PRP	Platelet-rich plasma
RDX	Radixin
RCT	Randomized controlled trial
SHA	Sodium hyaluronic acid
SII	Systemic immune-inflammation index
SOD	Superoxide dismutase
SS	Stabilization splint
TAC	Total antioxidant capacity
TLR4	Toll-like receptor 4
TMD	Temporomandibular disorders
TMJ	Temporomandibular joint
TMJ-ID	Temporomandibular joint internal derangement
TNF	Tumour necrosis factor
